# Supplementary material for: A Likelihood Approach for Real-Time Calibration of Stochastic Compartmental Epidemic Models
Source: PLoS Comput Biol. 2017 Jan 17;13(1):e1005257. doi: 10.1371/journal.pcbi.1005257 (PMC5240920; doi:10.1371/journal.pcbi.1005257)
Supplement: S1 File — (TAR.GZ) [file pcbi.1005257.s014.tar.gz › HSPH_Online-SI-Revision/MSS10/n100-extreme/n100-extreme_NEW_table4.pdf]

1	R0	{2.76052, 16.3913, 0.1}	{2.48103, 13.7658, 2.}	{{2.48103, 13.7658, 2.}, {2.48103, 13.7658, 2.}}	{10.1246, 16.0179, 1900.}	inf	11 577.	8888.24	{8832.32, 8938.15}	23.225	0.114786	newly	4336.	3858.17	{3778., 3923.}	11.0201	0.0507482	Reff	0.335762	0.368477	{0.366897, 0.369834}	9.74337	ar	92 090.	89 238.2	{89 116., 89 340.}	3.09677
2	R0	{2.18202, 15.1734, 3.}	{2.24606, 16.668, 3.}	{{2.24606, 16.668, 3.}, {2.24606, 16.668, 3.}}	{2.9351, 9.85011, 0.}	inf	9523.	9802.18	{9735.37, 9864.08}	2.93162	0.012544	newly	3796.	3701.66	{3631., 3763.}	2.52266	0.0111319	Reff	0.491915	0.500082	{0.498672, 0.501571}	1.66038	ar	84 264.	84 723.4	{84 549., 84 894.}	0.545203
3	R0	{2.57891, 12.5971, 1.}	{2.5898, 12.9574, 2.}	{{2.5898, 12.9574, 2.}, {2.5898, 12.9574, 2.}}	{0.422351, 2.86046, 100.}	inf	6810.	6819.29	{6769.52, 6860.45}	0.44181	0.00191603	newly	3181.	3076.51	{3013., 3134.}	3.28482	0.0145542	Reff	0.320791	0.321905	{0.320699, 0.32313}	0.387695	ar	90 302.	90 291.3	{90 178., 90 394.}	0.074273
4	R0	{2.5856, 9.28508, 0.1}	{2.59957, 9.40531, 0.09}	{{2.59708, 9.39342, 0.}, {2.59708, 9.39342, 0.}}	{0.540177, 1.2949, 184.}	inf	4198.	4026.89	{3951.89, 4044.96}	5.28489	0.023239	newly	2428.	2317.46	{2248., 2369.}	5.44234	0.0241538	Reff	0.287622	0.293599	{0.292823, 0.295261}	2.33187	ar	90 312.	90 220.	{90 103., 90 274.}	0.176023
5	R0	{2.4406, 12.1341, 1.}	{2.44101, 12.0941, 0.}	{{2.44101, 12.0941, 0.}, {2.44101, 12.0941, 0.}}	{0.0168666, 0.3296, 100.}	inf	8911.	8569.76	{8513.67, 8622.12}	3.82941	0.0169633	newly	4440.	4172.25	{4100., 4236.}	6.03041	0.0270435	Reff	0.386079	0.394473	{0.393132, 0.395773}	2.17438	ar	88 597.	88 205.1	{88 065., 88 325.}	0.442329
6	R0	{2.72155, 15.4094, 2.}	{2.75174, 15.3312, 0.01}	{{2.7499, 15.3124, 0.}, {2.7499, 15.3124, 0.}}	{1.10912, 0.73868, 99.5}	inf	11 194.	11 244.1	{11 162.5, 11 291.6}	0.612752	0.00254825	newly	4357.	4433.74	{4344., 4510.}	1.88662	0.00807965	Reff	0.34629	0.348702	{0.347154, 0.350745}	1.01644	ar	91 682.	91 756.2	{91 620., 91 842.}	0.111123
7	R0	{2.63781, 14.3935, 0.1}	{2.6365, 15.4074, 0.}	{{2.6365, 15.4074, 0.}, {2.6365, 15.4074, 0.}}	{0.0497021, 7.0439, 100.}	inf	9306.	8564.86	{8516.37, 8620.95}	7.96412	0.0360484	newly	3953.	3361.36	{3289., 3427.}	14.9669	0.0704636	Reff	0.327906	0.347283	{0.345785, 0.348532}	5.90938	ar	91 192.	90 439.9	{90 319., 90 552.}	0.824765
8	R0	{2.82391, 16.104, 1.}	{2.93371, 17.1903, 1.}	{{2.93371, 17.1903, 1.}, {2.93371, 17.1903, 1.}}	{3.88808, 6.74551, 0.}	inf	11 648.	12 377.1	{12 317., 12 432.4}	6.2592	0.0263634	newly	4404.	4380.51	{4310., 4456.}	1.18733	0.00517966	Reff	0.322067	0.3132	{0.311515, 0.314912}	2.75302	ar	92 664.	93 413.7	{93 311., 93 521.}	0.809074
9	R0	{2.80645, 14.6457, 0.1}	{2.36114, 11.6557, 1.}	{{2.36114, 11.6557, 1.}, {2.36114, 11.6557, 1.}}	{15.8673, 20.4153, 900.}	inf	11 375.	7487.99	{7409.35, 7546.4}	34.1715	0.181595	newly	4649.	3727.62	{3673., 3799.}	19.8189	0.0959681	Reff	0.325885	0.365954	{0.364455, 0.367636}	12.2953	ar	92 567.	88 056.8	{87 918., 88 173.}	4.87237
10	R0	{1.86213, 5.4271, 0.1}	{1.83544, 5.78475, 5.}	{{1.83544, 5.78475, 5.}, {1.83544, 5.78475, 5.}}	{1.43347, 6.59003, 4900.}	inf	1169.	1213.5	{1153.74, 1286.52}	4.53586	0.0191167	newly	1101.	1083.51	{1013., 1141.}	3.59764	0.0159089	Reff	0.480541	0.472836	{0.471484, 0.473858}	1.60344	ar	75 362.	75 280.4	{75 153., 75 401.}	0.149001
11	R0	{2.27349, 14.6338, 1.}	{2.40085, 15.146, 2.}	{{2.40085, 15.146, 2.}, {2.40085, 15.146, 2.}}	{5.60197, 3.50004, 100.}	inf	9305.	10 409.3	{10 337., 10 473.3}	11.8677	0.0486988	newly	3974.	4229.44	{4157., 4294.}	6.42778	0.0270134	Reff	0.44615	0.444631	{0.443052, 0.446265}	0.383232	ar	85 805.	87 511.7	{87 375., 87 657.}	1.98906
12	R0	{2.77261, 14.1894, 0.1}	{2.44101, 12.0941, 0.}	{{2.44101, 12.0941, 0.}, {2.44101, 12.0941, 0.}}	{11.9598, 14.7666, 100.}	inf	10 089.	6083.44	{6033.89, 6135.18}	39.7022	0.219709	newly	4154.	2945.23	{2885., 3003.}	29.0989	0.149402	Reff	0.306207	0.367361	{0.366058, 0.368533}	19.9716	ar	92 508.	87 920.5	{87 809., 88 034.}	4.95902
13	R0	{2.65402, 19.3935, 2.}	{1.98131, 11.1772, 1.}	{{1.98131, 11.1772, 1.}, {1.98131, 11.1772, 1.}}	{25.3468, 42.3662, 50.}	inf	15 461.	7180.96	{7093.64, 7262.13}	53.5543	0.333071	newly	4998.	3825.28	{3762., 3889.}	23.4638	0.116179	Reff	0.43088	0.485719	{0.484099, 0.48737}	12.7271	ar	91 184.	80 903.5	{80 705., 81 078.}	11.2745
14	R0	{1.75297, 4.45354, 0.1}	{1.71582, 3.97874, 1.}	{{1.71582, 3.97874, 1.}, {1.71582, 3.97874, 1.}}	{2.11921, 10.6612, 900.}	inf	1073.	806.042	{737.584, 875.045}	24.8796	0.125153	newly	1153.	950.77	{867., 1036.}	17.5395	0.0847868	Reff	0.522297	0.515809	{0.514618, 0.516969}	1.24221	ar	71 263.	70 763.	{70 605., 70 922.}	0.701697
15	R0	{1.99928, 7.1501, 0.1}	{1.96072, 6.67505, 4.}	{{1.96072, 6.67505, 4.}, {1.96072, 6.67505, 4.}}	{1.92892, 6.64403, 3900.}	inf	2594.	1907.77	{1844.26, 1970.63}	26.4545	0.133599	newly	1932.	1514.57	{1451., 1574.}	21.6061	0.105962	Reff	0.446419	0.451263	{0.450031, 0.452506}	1.08507	ar	79 602.	78 456.6	{78 305., 78 583.}	1.43897
16	R0	{2.67398, 10.3134, 1.}	{2.74056, 10.6768, 0.}	{{2.74056, 10.6768, 0.}, {2.74056, 10.6768, 0.}}	{2.48986, 3.52314, 100.}	inf	4744.	5657.43	{5612.3, 5699.57}	19.2545	0.076467	newly	2584.	2927.52	{2874., 2981.}	13.2941	0.0541673	Reff	0.275794	0.257628	{0.256461, 0.258856}	6.58691	ar	91 289.	92 317.3	{92 226., 92 402.}	1.12638
17	R0	{2.44634, 16.3226, 0.1}	{2.1245, 12.2645, 1.}	{{2.1245, 12.2645, 1.}, {2.1245, 12.2645, 1.}}	{13.1558, 24.8621, 900.}	inf	11 754.	7868.69	{7792.96, 7944.56}	33.0553	0.174295	newly	4470.	3846.7	{3753., 3905.}	13.9441	0.0652613	Reff	0.431632	0.457391	{0.455699, 0.458952}	5.9678	ar	88 710.	83 693.8	{83 495., 83 853.}	5.65456
18	R0	{2.02168, 10.1676, 0.1}	{1.93462, 9.38215, 4.}	{{1.93462, 9.38215, 4.}, {1.93462, 9.38215, 4.}}	{4.30613, 7.72501, 3900.}	inf	6854.	5540.52	{5484.62, 5599.44}	19.1636	0.0924107	newly	4020.	3452.37	{3389., 3507.}	14.1201	0.066152	Reff	0.515609	0.518817	{0.517671, 0.519887}	0.622181	ar	80 299.	78 091.6	{77 881., 78 284.}	2.74904
19	R0	{2.89969, 15.009, 0.1}	{2.52588, 12.2207, 0.}	{{2.52588, 12.2207, 0.}, {2.52588, 12.2207, 0.}}	{12.8912, 18.5773, 100.}	inf	11 247.	8288.43	{8223.11, 8343.9}	26.3054	0.132571	newly	4512.	3927.04	{3862., 3981.}	12.9645	0.0603395	Reff	0.292405	0.32944	{0.328025, 0.330864}	12.6659	ar	93 349.	90 268.3	{90 143., 90 376.}	3.30023
20	R0	{2.68933, 7.34928, 0.1}	{2.78624, 7.73801, 1.}	{{2.78624, 7.73801, 1.}, {2.78624, 7.73801, 1.}}	{3.60334, 5.28939, 900.}	inf	2277.	2986.85	{2944.86, 3025.9}	31.1747	0.117827	newly	1542.	1943.72	{1896., 1991.}	26.0519	0.10047	Reff	0.246316	0.235413	{0.234312, 0.236577}	4.42622	ar	91 574.	92 407.1	{92 338., 92 478.}	0.9098
21	R0	{2.15352, 9.28074, 0.1}	{2.10405, 8.58066, 3.}	{{2.10405, 8.58066, 3.}, {2.10405, 8.58066, 3.}}	{2.29732, 7.54334, 2900.}	inf	5169.	4419.19	{4362.85, 4481.46}	14.5059	0.0680868	newly	3172.	2872.42	{2807., 2925.}	9.44451	0.0431406	Reff	0.421315	0.427412	{0.426082, 0.428539}	1.44722	ar	83 845.	82 583.7	{82 439., 82 767.}	1.50434
22	R0	{2.21953, 9.04387, 0.1}	{2.13505, 8.31782, 0.}	{{2.13505, 8.31782, 0.}, {2.13505, 8.31782, 0.}}	{3.80628, 8.02813, 100.}	inf	4637.	3294.04	{3226.4, 3351.46}	28.9619	0.148553	newly	2827.	2172.82	{2112., 2221.}	23.1404	0.114383	Reff	0.397451	0.410996	{0.4095, 0.412296}	3.40792	ar	84 856.	82 844.8	{82 721., 82 955.}	2.37013
23	R0	{2.21987, 14.5347, 1.}	{2.11132, 12.644, 0.}	{{2.11132, 12.644, 0.}, {2.11132, 12.644, 0.}}	{4.88971, 13.0079, 100.}	inf	9287.	7044.25	{6969.95, 7110.76}	24.1494	0.120055	newly	3912.	3390.97	{3330., 3451.}	13.3188	0.0621144	Reff	0.465951	0.490519	{0.489085, 0.49206}	5.27269	ar	85 067.	82 097.7	{81 899., 82 268.}	3.49051
24	R0	{2.84268, 17.9659, 1.}	{2.315, 15.4658, 1.}	{{2.315, 15.4658, 1.}, {2.315, 15.4658, 1.}}	{18.5627, 13.9158, 0.}	inf	14 057.	9281.98	{9208.41, 9339.27}	33.969	0.180259	newly	4888.	3665.04	{3591., 3732.}	25.0196	0.125098	Reff	0.351242	0.396583	{0.395027, 0.398063}	12.909	ar	92 836.	87 627.2	{87 469., 87 773.}	5.61081
25	R0	{2.52706, 10.3679, 0.1}	{2.37615, 9.11994, 4.}	{{2.37615, 9.11994, 4.}, {2.37615, 9.11994, 4.}}	{5.97163, 12.0368, 3900.}	inf	5201.	4206.72	{4149.62, 4261.7}	19.117	0.0921635	newly	2743.	2510.54	{2447., 2565.}	8.47466	0.0385331	Reff	0.316085	0.320835	{0.319527, 0.32219}	1.50279	ar	89 623.	88 292.2	{88 171., 88 402.}	1.48488
26	R0	{2.62835, 19.1088, 1.}	{2.29055, 13.7413, 0.}	{{2.29055, 13.7413, 0.}, {2.29055, 13.7413, 0.}}	{12.8522, 28.0891, 100.}	inf	14 411.	10 932.7	{10 873.8, 10 992.}	24.1366	0.119972	newly	4701.	4832.63	{4761., 4902.}	2.82004	0.0120527	Reff	0.418354	0.44426	{0.442763, 0.445602}	6.19215	ar	90 908.	86 943.9	{86 785., 87 085.}	4.36062
27	R0	{2.76831, 19.4155, 2.}	{2.42687, 16.0321, 3.}	{{2.42687, 16.0321, 3.}, {2.42687, 16.0321, 3.}}	{12.3339, 17.4265, 50.}	inf	12 467.	10 201.3	{10 144.9, 10 250.4}	18.1733	0.0871087	newly	3937.	3878.53	{3819., 3942.}	1.65735	0.00727894	Reff	0.347063	0.359241	{0.357936, 0.360584}	3.50897	ar	92 175.	89 623.	{89 502., 89 744.}	2.76869
28	R0	{2.96609, 16.8903, 1.}	{2.52368, 12.5037, 0.}	{{2.52368, 12.5037, 0.}, {2.52368, 12.5037, 0.}}	{14.9157, 25.971, 100.}	inf	11 510.	7262.43	{7201.58, 7317.91}	36.9033	0.200003	newly	4186.	3389.04	{3339., 3437.}	19.0387	0.0917596	Reff	0.274037	0.340358	{0.338956, 0.341844}	24.2013	ar	94 024.	89 611.7	{89 490., 89 743.}	4.69276
29	R0	{2.17046, 14.39, 0.1}	{2.11292, 12.9719, 0.}	{{2.11292, 12.9719, 0.}, {2.11292, 12.9719, 0.}}	{2.65108, 9.85466, 100.}	inf	10 054.	8932.64	{8853.14, 9008.64}	11.1534	0.0513695	newly	4398.	4232.37	{4155., 4302.}	3.76603	0.0167128	Reff	0.504914	0.515222	{0.513549, 0.516884}	2.04149	ar	84 001.	82 552.4	{82 348., 82 712.}	1.72446
30	R0	{2.88772, 12.4647, 1.}	{2.9195, 12.5668, 2.76}	{{2.80569, 11.6897, 1.}, {2.93502, 12.6864, 3.}}	{1.78225, 2.31128, 176.}	inf	8175.	8015.41	{7502.04, 8138.39}	1.95219	0.00871376	newly	3775.	3662.35	{3602., 3724.}	2.98887	0.0132126	Reff	0.263591	0.271065	{0.268831, 0.274388}	2.83556	ar	93 273.	93 147.2	{92 563., 93 315.}	0.158095
31	R0	{2.46831, 8.14308, 0.1}	{2.41784, 8.02695, 4.}	{{2.41784, 8.02695, 4.}, {2.41784, 8.02695, 4.}}	{2.04466, 1.42615, 3900.}	inf	3768.	3115.58	{3065.11, 3158.71}	17.3149	0.0826076	newly	2494.	2051.51	{1992., 2094.}	17.7422	0.0849062	Reff	0.317474	0.326757	{0.325655, 0.327952}	2.92412	ar	88 762.	87 877.5	{87 770., 87 967.}	0.996496
32	R0	{1.93236, 7.38987, 0.1}	{2.03307, 8.59564, 1.}	{{2.03307, 8.59564, 1.}, {2.03307, 8.59564, 1.}}	{5.21151, 16.3165, 900.}	inf	3204.	4528.77	{4460.24, 4591.52}	41.3474	0.150261	newly	2353.	2994.09	{2937., 3048.}	27.2456	0.104585	Reff	0.482588	0.480804	{0.479376, 0.482172}	0.372558	ar	77 654.	79 933.3	{79 772., 80 095.}	2.93524
33	R0	{2.82251, 17.6954, 1.}	{2.31035, 12.8066, 3.}	{{2.31035, 12.8066, 3.}, {2.31035, 12.8066, 3.}}	{18.1455, 27.6276, 200.}	inf	15 003.	8589.48	{8523.81, 8656.06}	42.7482	0.242219	newly	5209.	4032.03	{3954., 4105.}	22.5949	0.111273	Reff	0.365938	0.447712	{0.446089, 0.449192}	22.3462	ar	92 745.	85 939.9	{85 808., 86 066.}	7.33746
34	R0	{1.87083, 12.3994, 1.}	{2.14288, 16.6365, 3.}	{{2.14288, 16.6365, 3.}, {2.14288, 16.6365, 3.}}	{14.5417, 34.1715, 200.}	inf	8229.	10 021.4	{9926., 10 112.1}	21.7821	0.0855733	newly	4286.	3941.05	{3870., 4015.}	8.0483	0.0364827	Reff	0.630488	0.683762	{0.6818, 0.685682}	8.44962	ar	76 282.	80 111.	{79 830., 80 409.}	5.01948
35	R0	{2.95779, 16.3003, 1.}	{2.45229, 12.2941, 0.91}	{{2.4534, 12.3139, 1.}, {2.4534, 12.3139, 1.}}	{17.0906, 24.5776, 9.}	inf	14 081.	9134.99	{8982.15, 9220.06}	35.1254	0.187952	newly	5272.	4361.13	{4284., 4429.}	17.2775	0.0824125	Reff	0.314472	0.382015	{0.379998, 0.384426}	21.478	ar	93 873.	88 822.7	{88 637., 88 962.}	5.37992
36	R0	{2.18718, 14.261, 3.}	{2.16437, 13.8995, 4.22}	{{1.98732, 12.0157, 4.}, {2.2143, 14.4309, 5.}}	{2.97761, 4.39295, 40.6667}	inf	9571.	10 411.4	{6882.11, 11 464.8}	21.1382	0.0909343	newly	4189.	4549.54	{3470., 4904.}	16.1771	0.0679805	Reff	0.488835	0.463785	{0.452805, 0.497455}	5.91213	ar	84 379.	84 982.7	{80 396., 86 386.}	2.78867
37	R0	{2.44912, 9.31118, 0.1}	{2.49321, 9.76482, 1.21}	{{2.49869, 9.80052, 1.}, {2.49869, 9.80052, 1.}}	{1.96401, 4.90299, 1110.}	inf	4364.	5003.1	{4987.96, 5122.46}	15.5477	0.0627674	newly	2540.	2831.23	{2769., 2911.}	12.0744	0.0494293	Reff	0.332493	0.322494	{0.320267, 0.323614}	3.1303	ar	88 442.	89 149.9	{89 095., 89 315.}	0.878214
38	R0	{2.05949, 5.72514, 0.1}	{2.0598, 5.9936, 4.}	{{2.0598, 5.9936, 4.}, {2.0598, 5.9936, 4.}}	{0.015208, 4.6892, 3900.}	inf	1447.	1380.38	{1329.94, 1430.53}	4.67762	0.0209658	newly	1229.	1158.64	{1106., 1201.}	5.82099	0.0262624	Reff	0.397749	0.399182	{0.398125, 0.400149}	0.364813	ar	81 587.	81 497.8	{81 391., 81 613.}	0.123525
39	R0	{1.75505, 8.52404, 0.1}	{1.85376, 9.90957, 3.}	{{1.85376, 9.90957, 3.}, {1.85376, 9.90957, 3.}}	{5.62426, 16.2544, 2900.}	inf	4589.	6773.69	{6682.95, 6867.53}	47.607	0.169083	newly	3152.	4143.24	{4052., 4237.}	31.448	0.118692	Reff	0.605615	0.599178	{0.59736, 0.600827}	1.06296	ar	70 925.	75 200.2	{75 009., 75 421.}	6.02776
40	R0	{2.24724, 13.7861, 1.}	{2.43143, 15.4278, 3.}	{{2.43143, 15.4278, 3.}, {2.43143, 15.4278, 3.}}	{8.19637, 11.9087, 200.}	inf	8230.	11 157.	{11 096.4, 11 215.9}	35.5652	0.132144	newly	3630.	4439.2	{4366., 4504.}	22.292	0.0873686	Reff	0.451088	0.416893	{0.415424, 0.418344}	7.58066	ar	85 257.	88 607.2	{88 437., 88 744.}	3.92954
41	R0	{1.77635, 8.62406, 0.1}	{1.85533, 9.28761, 1.}	{{1.85533, 9.28761, 1.}, {1.85533, 9.28761, 1.}}	{4.44607, 7.69417, 900.}	inf	4190.	6161.78	{6085.98, 6244.15}	47.0592	0.167469	newly	2830.	3913.93	{3829., 3989.}	38.3014	0.140775	Reff	0.557952	0.546175	{0.544596, 0.547571}	2.11059	ar	72 941.	76 467.5	{76 255., 76 676.}	4.83476
42	R0	{1.80397, 5.4932, 1.}	{1.81768, 5.68021, 1.}	{{1.81768, 5.68021, 1.}, {1.81768, 5.68021, 1.}}	{0.760022, 3.40431, 0.}	inf	1509.	1770.22	{1712.98, 1840.82}	17.3108	0.0691748	newly	1451.	1635.06	{1567., 1698.}	12.685	0.0516529	Reff	0.511444	0.510582	{0.509299, 0.511611}	0.196784	ar	73 232.	73 645.7	{73 506., 73 795.}	0.564903
43	R0	{2.83096, 16.7077, 0.1}	{2.14747, 10.7247, 0.}	{{2.14747, 10.7247, 0.}, {2.14747, 10.7247, 0.}}	{24.1435, 35.8097, 100.}	inf	13 176.	5264.65	{5189.41, 5330.87}	60.0436	0.398435	newly	4750.	2861.9	{2807., 2916.}	39.7495	0.220092	Reff	0.342093	0.429394	{0.427915, 0.430824}	25.5195	ar	92 740.	83 391.6	{83 230., 83 525.}	10.0802
44	R0	{2.73678, 11.6021, 1.}	{2.8186, 12.0293, 1.}	{{2.8186, 12.0293, 1.}, {2.8186, 12.0293, 1.}}	{2.98974, 3.68172, 0.}	inf	5400.	5840.77	{5794.28, 5883.64}	8.16247	0.0340693	newly	2650.	2755.71	{2697., 2812.}	3.98906	0.016933	Reff	0.263771	0.259233	{0.258017, 0.260531}	1.72028	ar	92 003.	92 573.8	{92 491., 92 674.}	0.620393
45	R0	{2.14235, 6.98601, 0.1}	{2.14326, 6.48739, 0.}	{{2.14326, 6.48739, 0.}, {2.14326, 6.48739, 0.}}	{0.0424843, 7.13742, 100.}	inf	1905.	2100.2	{2045.32, 2159.75}	10.2467	0.0422791	newly	1420.	1651.51	{1595., 1713.}	16.3035	0.0654387	Reff	0.389458	0.38544	{0.384151, 0.386593}	1.03174	ar	82 974.	83 247.7	{83 134., 83 358.}	0.329826
46	R0	{2.23409, 9.21289, 0.1}	{2.32678, 10.0266, 4.}	{{2.32678, 10.0266, 4.}, {2.32678, 10.0266, 4.}}	{4.14909, 8.83273, 3900.}	inf	5803.	7088.31	{7014.31, 7162.41}	22.149	0.0868752	newly	3539.	4012.34	{3955., 4083.}	13.375	0.05448	Reff	0.407766	0.394778	{0.392864, 0.396402}	3.1851	ar	85 163.	86 825.6	{86 666., 87 006.}	1.95224
47	R0	{2.12258, 8.15265, 0.1}	{2.13209, 8.2877, 0.36}	{{2.12684, 8.23415, 0.}, {2.13505, 8.31782, 1.}}	{0.448215, 1.65649, 388.}	inf	3887.	4049.19	{3938.97, 4139.66}	4.17873	0.0177061	newly	2681.	2693.77	{2626., 2758.}	1.75569	0.00759474	Reff	0.420441	0.418865	{0.417225, 0.420298}	0.3863	ar	82 690.	82 945.7	{82 781., 83 132.}	0.313472
48	R0	{2.71771, 10.4636, 0.1}	{2.74056, 10.6768, 0.}	{{2.74056, 10.6768, 0.}, {2.74056, 10.6768, 0.}}	{0.840716, 2.03712, 100.}	inf	6665.	6804.8	{6748.3, 6855.52}	2.09752	0.00900537	newly	3585.	3548.52	{3487., 3610.}	1.40921	0.00617362	Reff	0.28718	0.285764	{0.284288, 0.287295}	0.54523	ar	91 716.	91 871.9	{91 779., 91 960.}	0.170908
49	R0	{2.44369, 6.66477, 0.1}	{2.45956, 6.45922, 4.}	{{2.45956, 6.45922, 4.}, {2.45956, 6.45922, 4.}}	{0.649395, 3.08419, 3900.}	inf	1262.	1251.47	{1215.15, 1293.26}	1.88428	0.00825191	newly	921.	946.42	{911., 982.}	3.38111	0.0143786	Reff	0.285399	0.287511	{0.286459, 0.288295}	0.740177	ar	88 827.	88 802.6	{88 741., 88 861.}	0.0477332
50	R0	{2.77383, 17.9775, 0.1}	{2.88523, 18.2127, 0.}	{{2.88523, 18.2127, 0.}, {2.88523, 18.2127, 0.}}	{4.01604, 1.30809, 100.}	inf	11 601.	14 062.9	{14 003.4, 14 113.4}	21.2211	0.0835763	newly	3992.	4707.28	{4632., 4779.}	17.9178	0.0715515	Reff	0.332083	0.274389	{0.272912, 0.276044}	17.3732	ar	92 334.	94 327.2	{94 250., 94 419.}	2.15872
